# Malignant cancer may increase the risk of all-cause in-hospital mortality in patients with acute myocardial infarction: a multicenter retrospective study of two large public databases

**DOI:** 10.1186/s40959-023-00156-3

**Published:** 2023-01-21

**Authors:** Jianfeng Ye, Luming Zhang, Jun Lyu, Yidan Wang, Shiqi Yuan, Zhifeng Qin, Yu Liu, Tao Huang, Jinwei Tian, Haiyan Yin

**Affiliations:** 1 Department of Intensive Care Unit, The First Affiliated Hospital of Jinan University, Guangzhou, Guangdong Province China; 2 Department of Clinical Research, The First Affiliated Hospital of Jinan University, Guangzhou, Guangdong Province China; 3 Department of Cardiology, The Key Laboratory of Myocardial Ischemia, The Second Affiliated Hospital of Harbin Medical University, Chinese Ministry of Education, Harbin, Heilongjiang Province China; 4 Department of Neurology, The First Affiliated Hospital of Jinan University, Guangzhou, Guangdong Province China

**Keywords:** Acute myocardial infarction, Cancer, In-hospital mortality, Inverse probability of treatment weighing

## Abstract

**Background:**

Acute myocardial infarction (AMI) and cancer are diseases with high morbidity and mortality worldwide, bringing a serious economic burden, and they share some risk factors. The purpose of this study was to determine the effect of cancer on the all-cause in-hospital mortality of patients with AMI.

**Methods:**

This multicenter retrospective study analyzed patients with AMI from the Medical Information Mart for Intensive Care IV (MIMIC-IV) database and eICU Collaborative Research Database (eICU-CRD) in the United States. Patients were divided into two groups based on whether they had concomitant malignant cancer: cancer and noncancer groups. The outcome was all-cause in-hospital mortality. The association between the two groups and their outcomes were analyzed using Kaplan–Meier and Cox proportional-hazards regression models. Propensity score matching (PSM) and propensity score based inverse probability of treatment weighting (IPTW) were used to further adjust for confounding variables to verify the stability of the results.

**Results:**

The study included 3,034 and 5,968 patients with AMI from the MIMIC-IV database and the eICU-CRD, respectively. Kaplan–Meier survival curves indicated that the probability of in-hospital survival was lower in patients with cancer than in those without cancer. After adjusting for potential confounding variables using multivariable Cox proportional hazards regression, the risk of all-cause in-hospital mortality was significantly higher in the cancer than the noncancer group, and the HR (95% CI) values for the cancer group were 1.56(1.22,1.98) and 1.35(1.01,1.79) in the MIMIC-IV database and the eICU-CRD, respectively. The same results were obtained after using PSM and IPTW, which further verified the results.

**Conclusions:**

Among the patients with AMI, the all-cause in-hospital mortality risk of those with cancer was higher than those without cancer. Therefore, when treating such patients, comprehensive considerations should be made from a multidisciplinary perspective involving cardiology and oncology, with the treatment plan adjusted accordingly.

**Supplementary Information:**

The online version contains supplementary material available at 10.1186/s40959-023-00156-3.

## Background

Acute myocardial infarction (AMI) is a common acute and critical cardiovascular disease that is caused by acute coronary cavity occlusion, and results in a sharp reduction of blood supply and myocardial ischemic necrosis [1]. AMI can be classified into ST-segment elevation myocardial infarction (STEMI) and non-ST-segment elevation myocardial infarction (NSTEMI) according to the presence of two or more adjacent ST-segment elevation electrocardiograms at disease onset [1]. Despite the development of percutaneous coronary intervention (PCI) and thrombolytic therapy programs over the past 20 years and the large improvements in AMI prognoses [2, 3], the condition still has high morbidity and in-hospital mortality that seriously threaten public health and increase the global disease burden [4]. When AMI is associated with other diseases, the prognosis is often worse [5]. Although AMI and cancer seem to be two completely different diseases, there is considerable overlap in their pathogeneses in both epidemiology and at the cellular and molecular levels. For example, traditional risk factors such as age, sex, and smoking [6] can all promote the occurrence and development of atherosclerosis and cancer [7, 8]. Developments in medical technology and the continuous improvements in treatment methods have improved the survival time of cancer survivors [9]. Malignant cancer can accelerate atherosclerosis via different mechanisms such as inducing chronic inflammation and promoting endothelial damage [10]. Surviving cancer may not only increase the risk of AMI but also brings certain challenges to cardiovascular treatment.

Both cancer and its treatment are risk factors for AMI [11], while cardiovascular event occurrence can affect the quality of life of patients with cancer and increase their risk of short-term cancer mortality [12]. A previous study found that there was a higher risk of AMI in patients with cancer in the first 6 months of diagnosis compared with the non-cancer population [13]. The cancer type, stage, and type of treatment are all factors that contribute to an increased AMI risk. For example, study by some scholars has shown that radiation therapy to breast cancer increases the risk of ischemic heart disease [14]. And another study showed that patients with cervical cancer who received radiation or chemotherapy also had a significantly increased risk of myocardial infarction [15]. Patients with cancer have a higher mortality rate after STEMI [16], and arterial thromboembolism (including myocardial infarction and stroke) occurrence is the main cause of mortality in patients with cancer receiving chemotherapy [17]. These studies explored the risk of cardiovascular disease from cancer and its treatment, however, there are relatively few prognostic academic reports on this part of the population. The present study utilized two large free public databases—the Medical Information Mart for Intensive Care IV (MIMIC-IV) database and eICU Collaborative Research Database (eICU-CRD) to further explore the impact of cancer on the all-cause in-hospital mortality of patients with AMI.

## Methods

### Data source

MIMIC is a database of critical care medicine on the United States created by a team of critical care physicians, emergency physicians, and computer specialists at the Beth Israel Deaconess Medical Center (BIDMC), Massachusetts General Hospital, Massachusetts Institute of Technology (MIT), and the University of Oxford. The version we utilized is MIMIC-IV 1.0, which contains case information on patients admitted to an ICU or emergency room at BIDMC between 2008 and 2019 [18]. The eICU Collaborative Research Database (eICU-CRD) was made available by Philips Healthcare in partnership with the MIT Laboratory for Computational Physiology [19]. As a multicenter database, eICU-CRD contains data on more than 200,000 admissions to ICUs from 208 hospitals in the United States between 2014–2015.

Data extracted from the MIMIC IV and eICU-CRD databases do not require individual informed consent because research data is publicly available and all patient data are de-identified. These databases were approved by the Massachusetts Institute of Technology (Cambridge, MA) and Beth Israel Deaconess Medical Center (Boston, MA), and consent was obtained for the original data collection. The author (L.Z.) attended a series of courses offered by the NIH and was granted access to these databases after passing the required assessment (ID: 38601114).

### Population

**Fig. 1:**
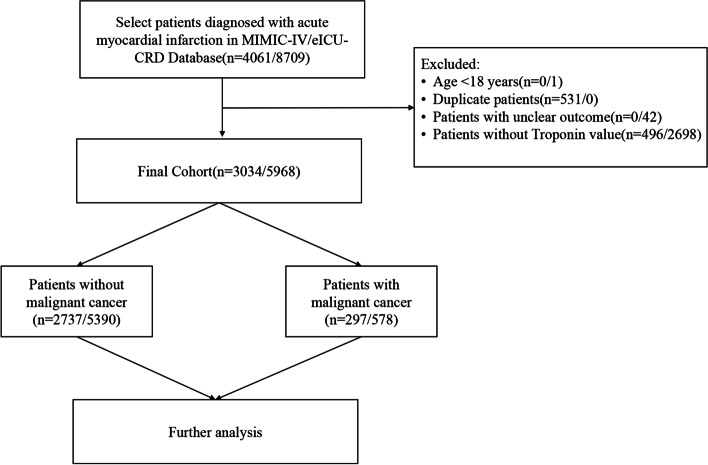
Inclusion and Exclusion Flowchart of the Study

All patients diagnosed with AMI in the MIMIC-IV database and the eICU-CRD were included. The exclusion criteria were patients younger than 18 years, duplicates, had unclear outcomes, and lacked troponin test results. Finally, 3,034 and 5,968 patients were included from the MIMIC-IV database and the eICU-CRD, respectively (Fig. 1).

### Data extraction

Missing data is a common phenomenon in the two databases. In order to achieve the consistency of variables as much as possible, we extracted the same variables with less than 20% missing values between the two databases. We use structured query language to extract data from the database. The data collected were age, gender, weight, Acute Physiological Score (APS), first care unit, intervention measures within 24 h of ICU admission, ventilator use, vasopressor use, dialysis use, whether they were undergoing PCI, whether CABG (coronary artery bypass grafting) was performed; main site of cancer; and comorbidities including congestive heart failure (CHF), hypertension, cerebrovascular disease (CD), chronic pulmonary disease (CPD), liver disease (LD), diabetes,renal disease (RD). Peak troponin: troponin t in MIMIC IV and troponin i in eICU-CRD; The results of the first laboratory examination after ICU admission were obtained for, white blood cell (WBC) (K/μL), hemoglobin (g/dL), platelets (K/μL), potassium (mEq/L), sodium (mEq/L), bicarbonate (mEq/L), creatinine (mg/dL), BUN (mg/dl), and glucose (mg/dL). Vital signs within 24 h of ICU admission were also obtained: heart rate, blood pressure (BP), respiratory rate, and temperature. Figure S1 shows the exact situation for continuous variables in both databases. The correlation matrix shows the Pearson's correlation between any two variables in the datasets [20].

The outcome assessed in this study was all-cause in-hospital mortality. Follow-up began with the admission of these patients and ended with their discharge or death.

### Statistical analyses

Patients with AMI were divided into two groups according to whether they had concomitant malignant cancer: cancer (not including patients with a previous history of cancer) and noncancer groups. After the Shapiro–Wilk test, the continuous variables in this study are all non-normal distributions, so the median and interquartile ranges were used to described. Kruskal–Wallis tests analyzed the differences in continuous variables. Categorical variables were described using counts and percentages, and χ2-test or Fisher’s exact test was used to test the distribution differences between groups. The association between the two groups and all-cause in-hospital mortality was analyzed using Kaplan–Meier and Cox proportional-hazards regression models. The log-rank test was conducted for nonparametric analysis to compare the survival distributions of the two groups. Cox multiple regression was used to control for confounding variables.

Propensity score matching (PSM) and inverse probability of treatment weighting (IPTW) were used to further verify the stability of the results and reduce the influence of data bias and confounding variables. When running PSM and IPTW with R software, missing variables are not allowed. So, the “mice” package of R software was used to account for missing covariate values using multiple imputation. In the PSM analysis, the cancer group included both the patients diagnosed with AMI and cancer after admission. Patients in the cancer group were matched with patients without cancer through nearest-neighbor matching at a 1:1 ratio. IPTW is an approximation method based on PS construction to deal with confounding variables, which applies the inverse of the propensity scores as weights to the original population and constructed two hypothetical populations. PSM reduces the sample size so that the populations of groups with the same PS have approximately equal sample sizes, while IPTW increases the sample size, which is similar to the formulation of a unified standard population, and adjusts the average level of the observed effects in the two groups according to the weights of confounding variables in the standard population, so as to eliminate the influence of the different distribution of internal confounding variables on the effect values between the two groups. Standardized mean differences (SMDs) before and after matching were used to determine whether PSM and IPTW reduced the differences in covariates between the two groups. Finally, variables that are still unbalanced and may be confounded by clinical judgment were further incorporated into the Cox multiple regression model for adjusting and verifying the results.

Further subgroup analyses were performed to assess the potential correction effects by age (< 55 years or ≥ 55 years), gender (male or female), PCI (no or yes), CABG (no or yes), ventilator use (no or yes), vasopressor use (no or yes). The potential interactions were evaluated by adding cross-product terms of groups with the above stratified variables to the model.

A two-sided *p* value < 0.05 was considered statistically significant. R software (version 4.0.3) was used for all statistical analyses. The R packages used included lattice, MASS, nnet, mice, survival, survminer, MatchIt, and ipw.

## Results

### Original population

In the MIMIC-IV database and the eICU-CRD, 3,034 and 5,968 patients, respectively, met the selection criteria and were included in this study. Dividing the patients with AMI into two groups according to whether or not they had malignant cancer resulted in the noncancer groups of the MIMIC-IV database and the eICU-CRD including 2,737 and 4,368 patients, respectively, and the cancer groups including 297 and 578. Table 1 lists the general condition, disease severity, comorbidities, and laboratory parameters of the patients. Patients were older in the cancer group than in the noncancer group in the MIMIC-IV database (73.00[65.00,80.00] vs. 70.00 [60.00–80.00] years) and in the eICU-CRD (75.00 [66.00, 82.75] vs. 65.00 [56.00, 76.00] years). In the MIMIC-IV database and eICU-CRD, male patients in the cancer group accounted for 63.0% and 57.6%, respectively. while the non-cancer group accounted for 62.7% and 63.4%; Those in the cancer group weighed less than those in the noncancer group in the MIMIC-IV database (72.00[62.50,87.60] kg vs 80.00[68.00,94.43] kg) and in the eICU-CRD (76.94 [64.46, 90.07]kg vs 83.00 [70.00, 98.30]). Disease severity scores were higher in the cancer group than in the noncancer group in the MIMIC-IV database (52.00[40.00,70.00] vs 43.00[30.00,62.00]) and in the eICU-CRD (38.00 [27.00, 52.50]vs 32.00[23.00,47.00]). Fewer patients in the cancer group underwent PCI than those in the noncancer group in the MIMIC-IV database (31.6% vs 40.8%) and in the eICU-CRD (19.4% vs 25.8%). The remaining baseline characteristics are listed in detail in Table 1.

**Table 1:** Baseline characteristics of the study population

	MIMIC-IV			eICU-CRD		
	Noncancer	Cancer	*P*-value	Noncancer	Cancer	*P*-value
	2737	297		5390	578	
Age(year)	70.00(60.00,80.00)	73.00(65.00,80.00)	0.004	65.00 (56.00, 76.00)	75.00 (66.00, 82.75)	< 0.001
Gender(%)			0.978			0.007
Male	1716(62.7)	187(63.0)		3418 (63.4)	333 (57.6)	
Female	1021(37.3)	110(37.0)		1972 (36.6)	245 (42.4)	
Weight(kg)	80.00(68.00,94.43)	72.00(62.50,87.60)	< 0.001	83.00 (70.00, 98.30)	76.94 (64.46, 90.07)	< 0.001
Severe score						
APS	43.00(30.00,62.00)	52.00(40.00,70.00)	< 0.001	32.00 (23.00, 47.00)	38.00 (27.00, 52.50)	< 0.001
First care unit(%)			< 0.001			0.097
NonCCU	751(27.4)	171(57.6)		2849 (52.9)	327 (56.6)	
CCU	1986(72.6)	126(42.4)		2541 (47.1)	251 (43.4)	
PCI(%)			0.003			0.001
no	1619(59.2)	203(68.4)		4002 (74.2)	466 (80.6)	
yes	1118(40.8)	94(31.6)		1388 (25.8)	112 (19.4)	
CABG(%)			< 0.001			0.961
no	2307(84.3)	285(96.0)		5220 (96.8)	559 (96.7)	
yes	430(15.7)	12(4.0)		170 ( 3.2)	19 ( 3.3)	
Ventilator(%)			0.549			0.124
no	1521(55.6)	171(57.6)		2461 (45.7)	244 (42.2)	
yes	1216(44.4)	126(42.4)		2929 (54.3)	334 (57.8)	
Vasopressor(%)			0.370			0.001
no	1872(68.4)	195(65.7)		4829 (89.6)	490 (84.8)	
yes	865(31.6)	102(34.3)		561 (10.4)	88 (15.2)	
Dialysis (%)			0.271			0.512
no	2685(98.1)	288(97.0)		5252 (97.4)	560 (96.9)	
yes	52(1.9)	9(3.0)		138 ( 2.6)	18 ( 3.1)	
Comorbidities(%)						
congestive heart failure						0.005
no	1255(45.9)	157(52.9)		4664 (86.5)	475 (82.2)	
yes	1482(54.1)	140(47.1)		726 (13.5)	103 (17.8)	
hypertension			0.105			< 0.001
no	1622(59.3)	191(64.3)		2331 (43.2)	204 (35.3)	
yes	1115(40.7)	106(35.7)		3059 (56.8)	374 (64.7)	
cerebrovascular disease			0.053			0.004
no	2366(86.4)	244(82.2)		4997 (92.7)	516 (89.3)	
yes	371(13.6)	53(17.8)		393 ( 7.3)	62 (10.7)	
chronic pulmonary disease			0.016			< 0.001
no	2119(77.4)	211(71.0)		4791 (88.9)	472 (81.7)	
yes	618(22.6)	86(29.0)		599 (11.1)	106 (18.3)	
renal failure			0.813			0.006
no	1885(68.9)	202(68.0)		4190(95.9)	444(93.1)	
yes	852(31.1)	95(32.0)		178(4.1)	33(6.9)	
liver disease			0.230			0.021
no	2537(92.7)	269(90.6)		5351 (99.3)	568 (98.3)	
yes	200(7.3)	28(9.4)		39 ( 0.7)	10 ( 1.7)	
diabetes			1.000			0.171
no	1628(59.5)	177(59.6)		3687 (68.4)	412 (71.3)	
yes	1109(40.5)	120(40.4)		1703 (31.6)	166 (28.7)	
Vital signs						
BP	59.00(51.00,66.00)	56.00(50.00,64.00)	0.003	119.00 (105.00, 136.00)	116.00 (102.00, 132.00)	0.002
Heart rate	66.00(59.00,76.00)	70.00(62.00,82.00)	< 0.001	78.00 (67.00, 90.00)	80.00 (68.00, 92.00)	0.110
Respiratory rate	12.00(10.00,14.50)	13.00(10.00,16.00)	< 0.001	18.00 (16.00, 22.00)	18.00 (16.00, 22.00)	0.449
Temperature	36.44(36.22,36.67)	36.44(36.33,36.67)	0.544	36.72 (36.50, 36.94)	36.61 (36.39, 36.89)	< 0.001
Laboratory tests						
Max troponin T/I	1.37 (0.36, 4.13)	0.79 (0.23, 1.93)	< 0.001	8.73 (1.69, 32.78)	5.72 (1.19, 17.96)	< 0.001
WBC(K/uL)	10.80(8.10,14.40)	9.70(7.40,14.40)	0.044	10.70 (8.30, 14.00)	10.30 (7.40, 14.20)	0.032
hemoglobin(g/dl)	11.60(9.80,13.40)	9.80(8.20,11.20)	< 0.001	13.40 (11.60, 14.90)	11.90 (10.10, 13.60)	< 0.001
platelet(K/uL)	208.00(162.00,262.00)	184.00(127.50,267.00)	0.001	222.00 (179.00, 274.00)	210.00 (162.00, 272.00)	0.001
potassium(mEq/L)	4.20(3.90,4.60)	4.30(3.90,4.70)	0.072	4.00 (3.70, 4.40)	4.10 (3.80, 4.50)	0.015
calcium(mEq/L)	8.60(8.20,9.00)	8.50(8.00,9.00)	0.006	8.90 (8.40, 9.30)	8.80 (8.30, 9.30)	0.015
sodium(mEq/L)	138.00(136.00,141.00)	137.00(134.00,140.00)	< 0.001	138.00 (135.00, 140.00)	138.00 (135.00, 140.00)	0.172
bicarbonate(mEq/L)	23.00(20.00,25.00)	22.00(18.00,25.00)	0.005	24.00 (22.00, 27.00)	24.00 (22.00, 27.00)	0.877
creatinine(mg/dl)	1.10(0.80,1.80)	1.20(0.90,1.70)	0.264	1.06 (0.85, 1.43)	1.15 (0.90, 1.59)	< 0.001
glucose(mg/dl)	139.00(111.00,193.00)	127.00(104.00,166.00)	< 0.001	138.00 (112.00, 193.00)	137.00 (115.00, 188.00)	0.975
Outcome						
All-cause in-hospital mortality (%)			< 0.001			< 0.001
no	2281(83.3)	196(66.0)		4938(91.6)	494(85.5)	
yes	456(16.7)	101(34.0)		452( 8.4)	84(14.5)	
Length of hospital stay (day)	6.98(3.65, 12.08)	8.82(3.88, 14.97)	< 0.001	3.43(2.15, 6.77)	4.37(2.52, 8.15)	< 0.001

**Fig. 2:**
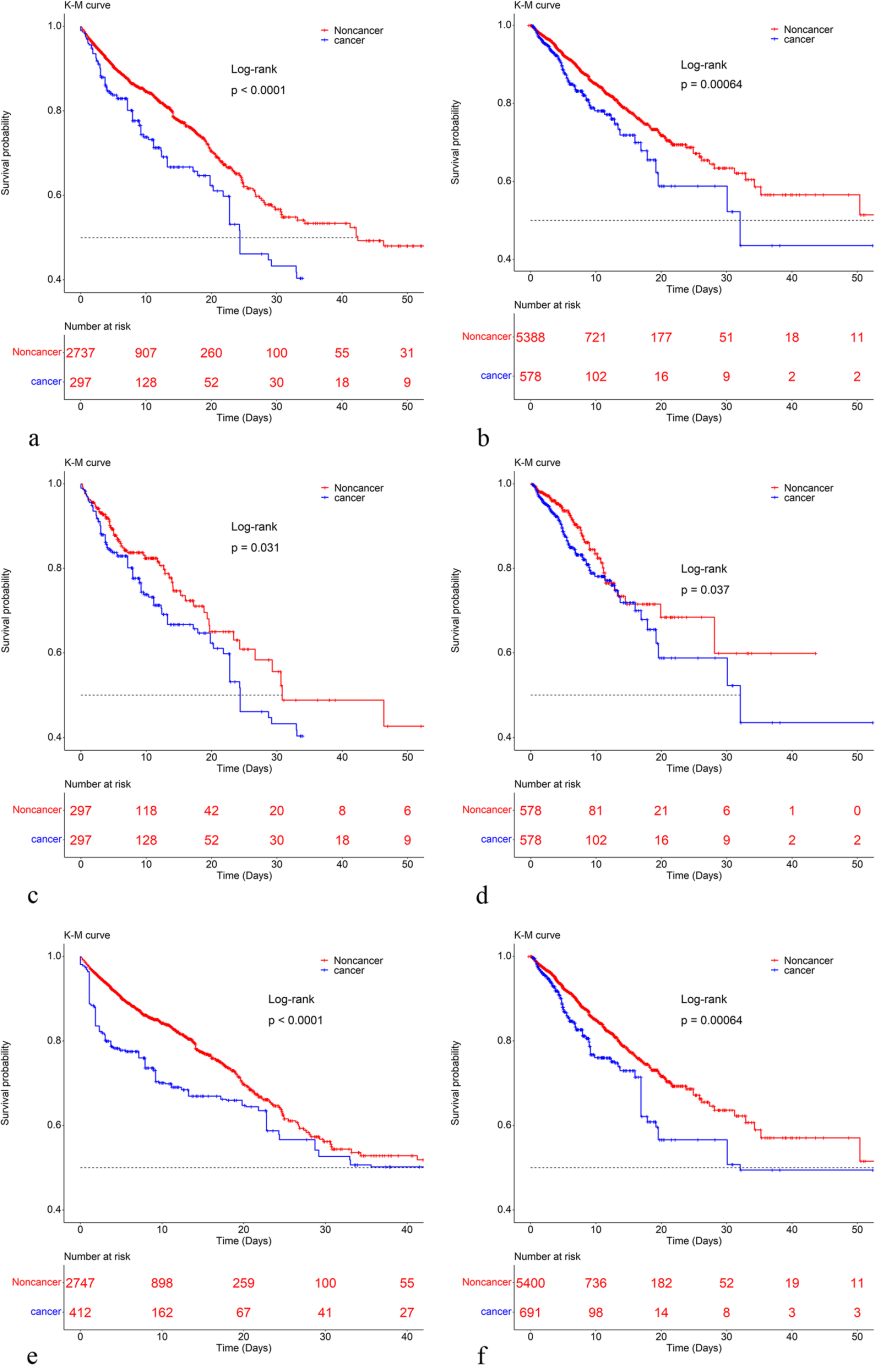
Kaplan–Meier survival curves between groups. **a**, **c**, and **e** are the original population, PSM population and IPTW population of the MIMIC database; **b**, **d**, and **f** are the original population, PSM population and IPTW population of the eICU-CRD database, respectively

The Kaplan–Meier survival curve indicated that the probability of in-hospital survival of patients in the cancer group was lower than that of patients in the noncancer group (Fig. 2). In this study, variables include age, gender, weight, APS, first care unit, ventilator use, vasopressor use, dialysis use, PCI, CABG; comorbidities (CHF, CD, CPD, LD, diabetes, and RD); laboratory indicators (troponin, WBC, hemoglobin, platelets, potassium, sodium, bicarbonate, creatinine, BUN, and glucose); vital signs within 24 h of admission to the ICU (heart rate, BP, respiratory rate, and temperature), the Pearson’s coefficients between the two random continuous variables were all less than 0.5 (Figure S2), indicating that there was no significant correlation between them. After adjusting for potential confounders by multiple Cox regression, the risk of all-cause in-hospital mortality was significantly higher in the cancer group than in the noncancer group, and the HR (95% CI) values for the cancer group were 1.56(1.22,1.98) and 1.35(1.01,1.79) in the MIMIC-IV database and the eICU-CRD, respectively, corresponding to 1.56- and 1.35- fold higher risks of all-cause in-hospital mortality in the cancer groups, respectively (Table 2).

**Table 2:** Analysis of the associations between all-cause in-hospital mortality and groups

	Non cancer	Cancer	
	HR(95%CI)	HR(95%CI)	*p*-value
MIMIC-IV			
Multivariate Model	Reference	1.56(1.22,1.98)	< 0.001
PSM	Reference	1.83(1.25,2.68)	0.001
IPTW	Reference	1.54(1.19,2.02)	0.001
eICU-CRD			
Multivariate Model	Reference	1.35(1.01,1.79)	0.039
PSM	Reference	1.54(1.04,2.29)	0.031
IPTW	Reference	1.51(1.20,2.03)	0.001

Confounders included age, gender, weight, APS, first care unit, ventilator use, vasopressor use, dialysis use, PCI, CABG; comorbidities (CHF, CD, CPD, LD, diabetes, and RD); laboratory indicators (troponin, WBC, hemoglobin, platelets, potassium, sodium, bicarbonate, creatinine, BUN, and glucose); vital signs within 24 h of admission to the ICU (heart rate, BP, respiratory rate, and temperature)

Cox proportional hazards regression models were used to calculate hazard ratios (HRs) with 95%

### Propensity score matching and inverse probability of treatment weighing

It can be observed in Table 1 that many variables such as age, weight, disease severity score, and PCI were all considerably unbalanced. However, after PSM these were all well balanced. The Kaplan–Meier survival curve indicated the same trend as the original population (Fig. 2). However, there were still some variables that differed between the groups (Table S1, Table S1,2). These variables were then again subjected to the Cox multiple regression, and the final results were consistent with those for the original population. In the MIMIC-IV database and the eICU-CRD, the all-cause in-hospital mortality risks of patients in the cancer groups were 1.83- and 1.54-fold higher, respectively, than those in the noncancer groups (Table 2).

The virtual populations obtained from the IPTW data set using multiple logistic regression were well balanced in the two databases. The results of the Kaplan–Meier survival curve were also consistent with the original population and the population obtained after matching; that is, the probability of all-cause in-hospital mortality was lower in the cancer than in the noncancer group (Fig. 2). These virtual populations were again subjected to Cox multiple regression, and a trend similar to that of the population is obtained. In the MIMIC-IV database and the eICU-CRD, the all-cause in-hospital mortality risks of the cancer groups were 1.54- and 1.51- fold higher than those of the noncancer groups, respectively (Table 2).

### Subgroup analyses

**Fig. 3:**
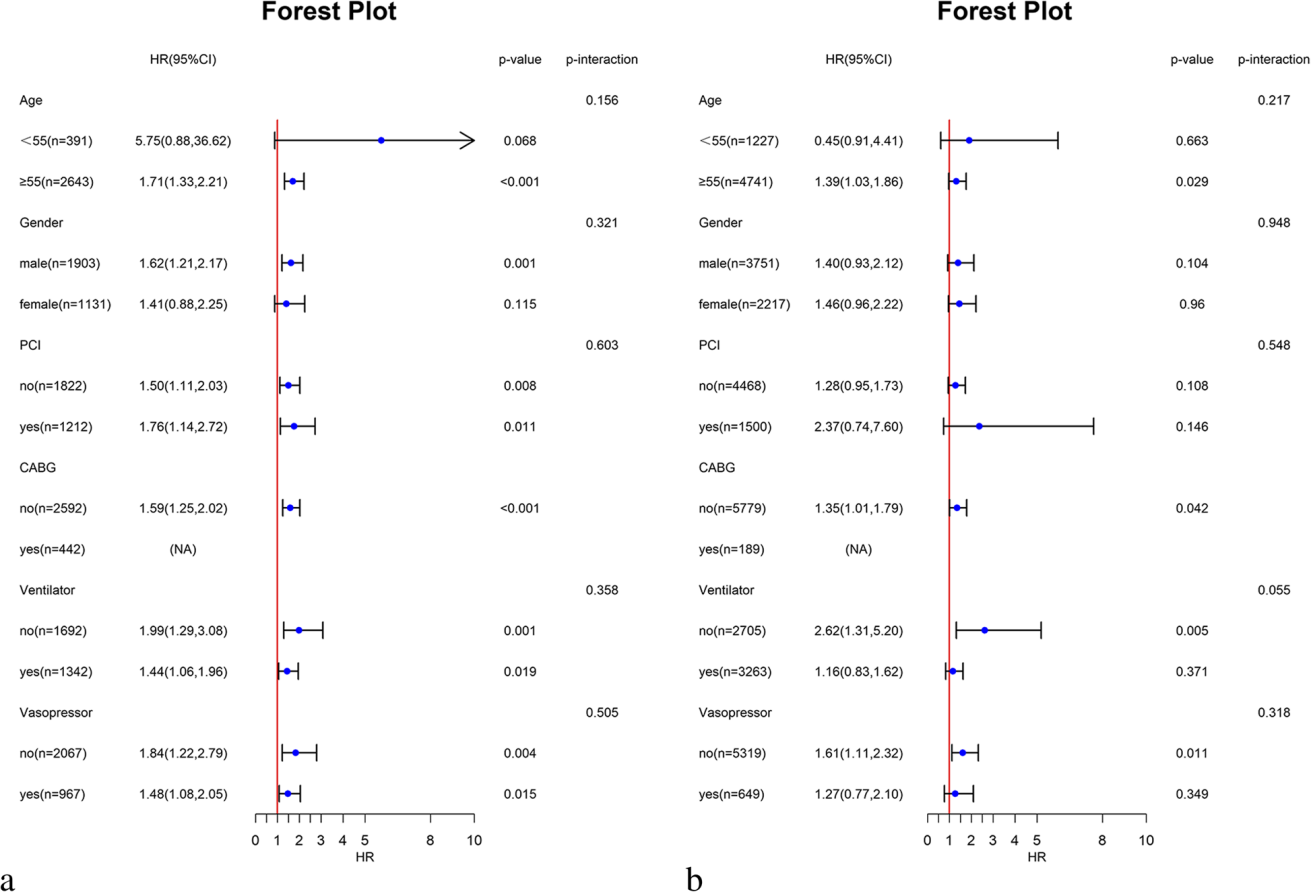
Subgroup analysis of relationship between groups and all-cause in-hospital mortality. **a**: MIMIC database; **b**: eICU-CRD database

There was no significant interaction between the groups in the two databases (Fig. 3).

## Discussion

In this study, a multicenter study based on population samples from two large public databases (MIMIC-IV and eICU-CRD), after adjusting for multiple confounders, cancer may increase the all-cause in-hospital mortality risk of patients with AMI by 1.56- and 1.35-fold, respectively, compared with those without cancer. And the two databases contain patients in different periods, which shows that cancer has always been a serious challenge to patients with AMI, whether in the present or in the past. In addition to this, PSM and IPTW were also used to further balance the confounding variables to verify the results, and improve their stability and credibility.

The above results may be explained by the patients with malignant cancer being older than those without cancer (like in our study population), whether data were obtained from the MIMIC-IV database or the eICU-CRD, and the median age of AMI groups with cancer being higher than in the groups without cancer. Age is not only an important risk factor for AMI but also for tumors [21, 22]. Old age is often accompanied by more complications and atypical clinical symptoms. Only 30.3% of cancer patients had chest pain and 44% had dyspnea at AMI, and the incidence rates of these symptoms were lower in NSTEMI than in STEMI [23]. The management of elderly patients with cancer was also often more complex, involving a variety of treatment methods that all bring certain difficulties to timely AMI diagnoses and PCI implementation [24]. Furthermore, the hematology of patients with cancer, such as anemia and hemolysis due to impaired red blood cell production, will further increase the risks of ischemia and bleeding. Our research results also indicated that the hemoglobin level in patients with cancer from the two databases was lower than that in the noncancer group. When AMI occurs in patients with cancer, these patients therefore often receive conservative treatment. Our results further indicated that fewer patients with cancer undergo PCI surgery. Some scholars have suggested that cancer is an independent risk factor for major adverse cardiovascular events after AMI such as revascularization and massive bleeding [25]. Cancer is also an independent predictor of adverse cardiovascular events in patients undergoing PCI [26].

Cardio-Oncology is a new field that is constantly being explored, which is to solve the intricate intersection between the two major causes of death in human beings [27]. For patients with AMI complicated by cancer, clinicians need to provide patients with a comprehensive multidisciplinary approach to individualized treatment before and during initial treatment. In the treatment, clinicians can try to apply the emerging and advantageous PCI optimization methods to AMI in patients with cancer [28], but it is worth noting that there is still a long way to go, and a lot of clinical research and data support are needed.

## Strengths and limitations

The greatest strengths of this study were undoubtedly long-time span, large sample, and the use of multicenter data from the MIMIC-IV database and the eICU-CRD. Moreover, PSM and IPTW methods were adopted to further balance confounding variables and verify the results, making them robust, reliable, and extrapolative. However, this study also had some inevitable limitations. First, it was a retrospective study. Second, due to the limitations of the database, this article only focused on patients with AMI who were still accompanied by cancer during hospitalization, excluding patients with a previous history of cancer. Third, we were unable to analyze the type and stage of cancer, whether it was metastatic, and the treatment the patients were receiving. Fourth, we could not know the exact time when the patient was diagnosed with AMI. Fifth, it is difficult to obtain information on patients’ medication. Also because of the database, we could only investigate the short-term mortality risk of patients, and so further research is needed into the long-term prognostic impact of cancer on patients with AMI.

## Conclusions

Among patients with AMI, the all-cause in-hospital mortality risk of patients with cancer was higher than that of those without cancer. When treating such patients, comprehensive considerations should be made from a multidisciplinary perspective involving cardiology and oncology, with the treatment plan adjusted accordingly.

## Supplementary Information

**Additional file 1.**

**Additional file 2.**

**Additional file 3.**

## Data Availability

The data were available on the MIMIC-IV website at https://mimic-iv.mit.edu/. The data were available on the eICU Collaborative Research Database at https://eicu-crd.mit.edu/
