# Supplementary figures and images for: Malignant cancer may increase the risk of all-cause in-hospital mortality in patients with acute myocardial infarction: a multicenter retrospective study of two large public databases
Source: Cardiooncology. 2023 Jan 21;9:6. doi: 10.1186/s40959-023-00156-3 (PMC9862556; doi:10.1186/s40959-023-00156-3)

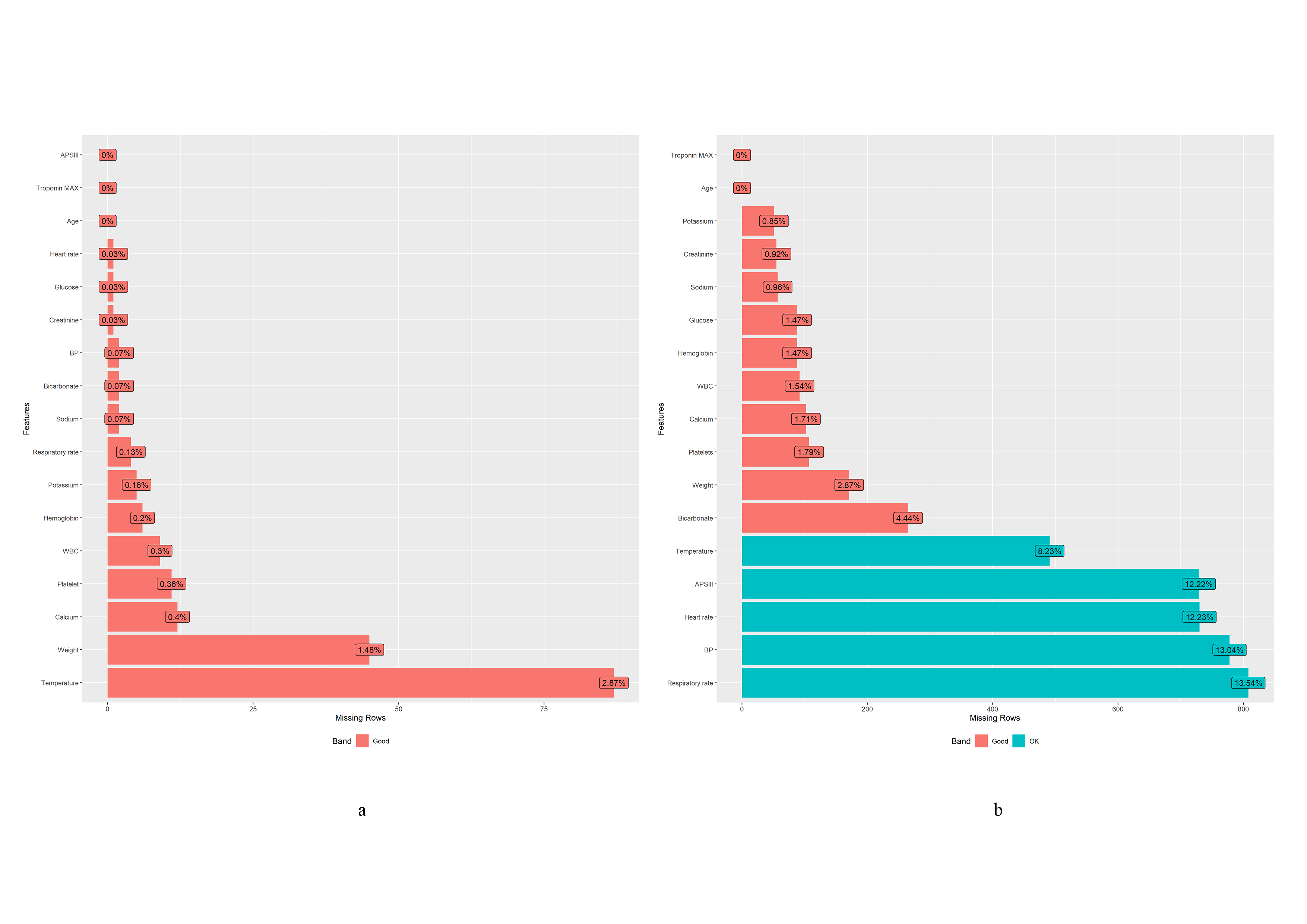

Supplement: Supplementary file 1 — Additional file 1. [file 40959_2023_156_MOESM1_ESM.zip › FigureS1.tif]

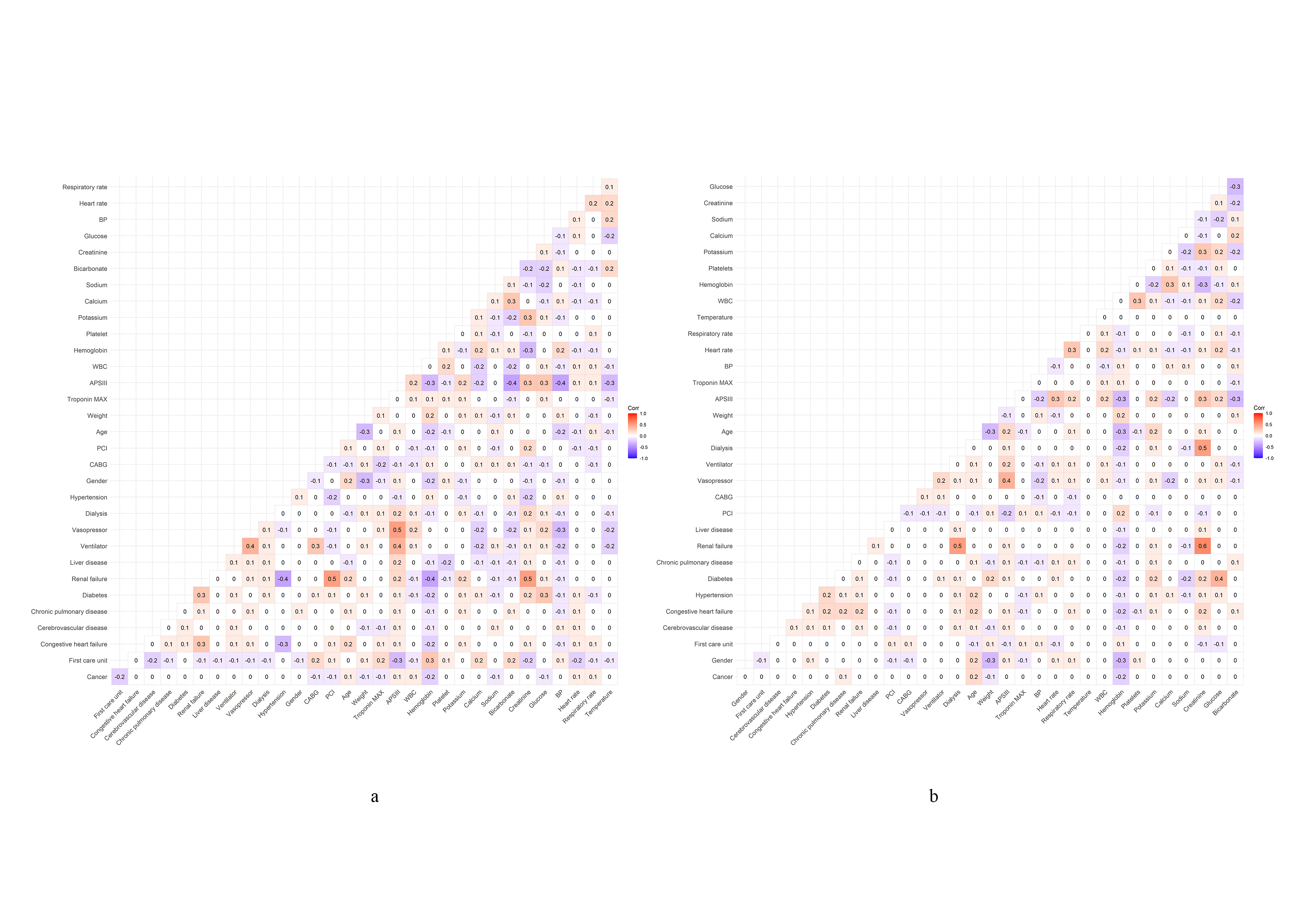

Supplement: Supplementary file 1 — Additional file 1. [file 40959_2023_156_MOESM1_ESM.zip › FigureS2.tif]
